# Psychoactive substances use and associated factors among Axum university students, Axum Town, North Ethiopia

**DOI:** 10.1186/1471-2458-13-693

**Published:** 2013-07-30

**Authors:** Measho Gebreslassie, Amsalu Feleke, Tesfahun Melese

**Affiliations:** 1 Department of Health Service Management and Health Economics, Institute of Public Health, College of Medicine and Health Sciences, University of Gondar, Gondar, Ethiopia; 2 Department of Health Informatics, Institute of Public Health, College of Medicine and Health Sciences, University of Gondar, Gondar, Ethiopia

**Keywords:** Psychoactive substance, Prevalence, University students, Axum

## Abstract

**Background:**

The use of substances such as alcohol, khat leaves and tobacco have long been recognized as one of the leading causes of human suffering and become one of the rising major public health and socio-economic problems worldwide. Even though substances use occurs in all segments of all societies, it is more spreading in an alarming rate among the young generation. This study aimed to establish the prevalence and associated factors of substances use among undergraduate students in Axum University.

**Methods:**

Institution based quantitative cross sectional study design was conducted among Axum University students in April 2012. A sample of 764 students was selected by using multi- stage sampling technique. Data were collected using pre- tested self- administered questionnaires. The data were cleaned, coded, entered into EPI-INFO version 3.5.1 and transferred and analysed using SPSS computer soft ware package version 20.

**Results:**

The lifetime prevalence of khat chewing, alcohol drinking and cigarette smoking among the study participants were 28.7%, 34.5% and 9.5% respectively. Similarly, the current prevalence of khat chewing, alcohol drinking and cigarette smoking were 27.9%, 32.8% and 9.3% respectively. The commonest reasons for khat, alcohol and cigarette using were to keep alert while reading 40.6%, for relaxation 65.5% and to relief stress 37.7% respectively. Having peer friends who chew khat was strongly and positively associated with khat use [AOR: 10.18, 95%CI: (5.59, 18.54)].Family members and peer friends alcohol use were strongly associated with alcohol drinking [AOR: 2.61, 95%CI: (1.56, 4.34) and [AOR: 14, 95%CI: (8.09, 24.24)] respectively. Ever alcohol use was strongly associated with cigarette smoking [AOR: 6.54, 95%CI: (2.66, 16.05)].

**Conclusion:**

This study revealed that psychoactive substances use became an urgent problem among undergraduate university students. Universities need to monitor and teach their students with special focus on fresh man students, about the health risks and socioeconomic problems associated with psychoactive substances use.

## Background

Use of substances such as alcohol, khat leaves (Catha edulis) and tobacco have long been recognized as one of the leading causes of human suffering and become one of the rising major public health and socio-economic problems worldwide [1-3]. Recent trends indicate that the use of substances have dramatically increased particularly in developing countries [1,4]. The use of alcohol, tobacco and other substances constitutes one of the most important risk–taking behaviors among adolescents and young adults in secondary schools and colleges with consequent physical and /or mental health complications [4,5]. Despite worldwide concern and education about psychoactive substances, many adolescents have limited awareness of their adverse consequences [5].

The transition from high school to college is a critical developmental period commonly associated with escalations in a range of health-risk behaviors including alcohol, tobacco and illicit drug use [6]. Substances use occurs in all segments of all societies especially among college students, which results in decreased work, decreased academic performance, increased risk of contracting HIV and other sexually transmitted diseases, accidents, intoxication while working, absenteeism, violent crime, theft and other psychiatric disorders such as lethargy, hopelessness and insomnia [4,7].

During the transition to college, young people encounter many new sources of stress, including separation from family, sharing close living quarters with strangers, the formation of new social groups, intense academic pressures and the balancing of social engagements with academic and other life responsibilities [8]. In 2006 there were around 190 million drug abusers around the globe, which accounts for 3.1%of the world population or 4.3 %of the population aged 15 years and above [9]. A study conducted among college students in Eldoret, Western Kenya showed that the lifetime prevalence rate of any substances use was 69.8%; Lifetime prevalence rate of alcohol and cigarette use was 51.9% and 42.8% respectively [10].

Substances use is one of the most burning and growing public health problems in Ethiopia, as in many developing countries; alcohol, khat and tobacco are the most frequently used substances. However, hard drugs such as heroin and cocaine are rarely used [4,11]. In Ethiopia, khat is commonly used for stimulation and social recreation. A significant number of students consume khat to be alert and wakeful at night, especially during examination periods [9]. Availability of substances, age, gender, having friends and families who use substances were the commonly mentioned factors that influence substances use [4,5,7].

Though psychoactive substances use have become common practices among university students in Ethiopia, few studies have assessed the magnitude and associated factors. The objective of this study was to assess the prevalence of psychoactive substances use and associated factors among Axum university students.

## Methods

This study was conducted from April to June 2012 to assess psychoactive substances use and associated factors among students in Axum University, Ethiopia. Axum University is found in Axum Town, Central Tigray zone, Northern Ethiopia. It is one of the newly established public higher education institutions in Ethiopia located about 1010 km far from Addis Ababa, the Ethiopian Capital city. Axum enshrines one of the most impressive archeological and historical areas in the world. The University has a total of 9,200 students enrolled in 42 academic departments. The study utilized institution-based cross-sectional study design with quantitative data collection method. The study population included all regular undergraduate students attending at Axum University. Students who were seriously ill and absent during the study period were excluded from the study.

The sample size was calculated using single population proportion formula with the following assumptions; prevalence of substances use 17.5%, which was obtained from research conducted among college students in North West Ethiopia [12]. Using 4% margin of error at 95% confidence level, the sample size was 764 after considering 10% non response rate and design effect of two.

The sample was obtained using multi-stage sampling technique. During the first stage, eight departments were selected among 42 departments by using simple random sampling. In the second stage, departments, in each field of study, were further stratified by their years of study, assuming that their years of study and duration of stay in the campus would affect psychoactive substances use among the students. Finally, the total sample size was distributed proportionally to the selected departments based on total number of students in each year of study. Individual students from each stratum were selected by using simple random sampling technique using computer generated random numbers. The sampling frame was students’ identification number in their respective batch or years of study.

The operational definition of some words and phrases are stated as follow:

Life time prevalence of substances use is: the proportion of students who had ever used substance in their life time; current prevalence of substances use is: the proportion of students who were used substance within 30 days preceding the study; and Ever substances used are: Students who self-reported that they had used the substances on one or more than one occasion/s.

The dependent variables were alcohol drinking, khat chewing and cigarette smoking while the following factors were included in the model as independent variables: socio- demographic characteristics (age, sex, marital status, religion and ethnicity), years of study, residence during school age, peer group substances use, family member substances use and knowledge on the health risks of substances use.

The questionnaires were prepared by reviewing relevant literatures [4,10,12,13]. Pre- test was done on 10% of the subjects at Gondar University. Data were collected by pre-tested, pre-coded and self-administered questionnaires. The collected data were cleaned, coded, entered into EPI-INFO version 3.5.1 software and transferred and analysed using SPSS computer soft ware package version 20. Summary statistics of socio demographic variables were presented using frequency tables and graphs. Bivariate analysis was done and variables with p-value less than 0.2 were included in the multiple logistic regression analysis. Odds ratio and 95% confidence intervals were also computed along with the corresponding p-value.

The study was reviewed and approved by Institution Research Review Boards, Institute of Public Health at the University of Gondar and Axum University. The purpose and the importance of the study were explained and written consent was obtained from each participant. Moreover, confidentiality of the information was assured by using anonymous questionnaires and by keeping the data in a secured place.

## Results

Out of 764 students participated in the study, 756 completed the questionnaires making the response rate of 98.7%. Among the study subjects, 444 (58.8%) were males, 594 (78.6%) were in the age group of 20–24 and the mean age of the respondents was 22.3 years (SD ± 2.2 years). Four hundred twenty two (55.8%) of the samples were Orthodox Christians and 333 (44%) were Tigrie in Ethnic group. Most of the students 672 (88.9%) were single. With regard to the area of original residence, 450 (59.5%) were from urban back ground (Table 1).

**Table 1:** Socio-demographic characteristics of Axum university students by sex (n = 756), April 2012

**Characteristics**	**Sex**		**Total n (%)**
	**Male n (%)**	**Female n (%)**	
**Age group( years)**			
15-19	24 (5.4)	26 (8.3)	50 (6.6)
20-24	342 (77.0)	252 (80.8)	594 (78.6)
25-30	78 (17.6)	34 (10.9)	112 (14.8)
total	444 (100)	312(100)	756(100)
**Religion**			
Orthodox	263 (59.2%)	159 (51.0%)	422 (55.8%)
Muslim	110 (24.8%)	85 (27.2%)	195 (25.8%)
Protestant	65 (14.6%)	60 (19.2%)	125 (16.5%)
Others	6 (1.4%)	8 (2.6%)	14 (1.9%)
**Ethnicity**			
Tigrie	193 (43.5)	140 (44.9)	333 (44)
Amhara	142 (32.0)	90 (28.8)	232 (30.7)
Oromo	69 (15.5)	52 (16.7)	121 (16)
SNN	40(9.0)	30(9.6)	69(9.1)
**Marital status**			
Single	403(90.8)	269(86.1)	672(88.9)
Married	26(5.8)	32(10.3)	58(7.7)
Divorced	6(1.4)	8(2.6)	14(1.9)
Others	9(2.9)	3(1.0)	12(1.6)
**Original residence**			
Urban	261(58.8)	189(60.6)	450(59.5)
Rural	183(41.2)	123(39.4)	306(40.5)
**Year of study**			
First year	155(34.9)	123(39.4)	278(36.8)
Second year	152(34.2)	95(30.4)	247(32.7)
Third year	119(26.8)	78(25.0)	197(26)
Fourth year	18(4.1)	16(5.1)	34(4.5)
**Faculty**			
Health	22(5.0)	20(6.4)	42(5.6)
Social	44(9.9)	27(8.7)	71(9.4)
Business and economics	98(22.1)	84(26.9)	182(24.1)
Natural	128(28.8)	67(21.5)	195(25.8)
Engineering	152(34.2)	114(36.5)	266(35.2)

The overall life time and current prevalence of psychoactive substances use among the study subjects were 45.9% and 44.8% respectively. The life time prevalence of khat use, alcohol drinking and cigarette smoking among the study participants were 28.7%, 34.5% and 9.5% respectively. Similarly, the current prevalence of khat chewing, alcohol drinking and cigarette smoking were 27.9%, 32.8% and 9.3% respectively. The minimum age for khat chewing, alcohol drinking and cigarette smoking were 14 years, 10 years and 16 years respectively. At the same time, the mean age at which the respondents started khat chewing, alcohol drinking and cigarette smoking were 20.1 years (SD ± 2.75 years), 19.5 years (SD ± 2.2 years) and 20.5 years (SD ± 2.2 years) respectively (Table 2).

**Table 2:** Life time and current prevalence of psychoactive substances use among Axum university students by sex, April 2012

**Characteristics**		**Sex**		
**Substances use**		**Male n (%)**	**Female n (%)**	**Total n (%)**
Ever used	Yes	251(56.5)	96(30.8)	347(45.9)
	No	193 (43.5)	216(69.2)	409 (54.1)
Current users	Yes	245 (55.2)	94(30.1)	339 (44.8)
	No	199 (44.8)	218(69.9)	417(55.2)
**Khat chewing**				
Ever chewed	Yes	162 (36.5)	55(17.6)	217 (28.7)
	No	282 (63.5)	257(82.4)	539 (71.3)
Chewed khat in the last 12 months	Yes	162 (36.5)	55(17.6)	217 (28.7)
	No	282 (63.5)	257(82.4)	539 (71.3)
Current chewers	Yes	158 (35.6)	53(17)	211 (27.9)
	No	286 (64.4)	259(83)	545 (72.1)
**Drinking alcohol**				
Ever drunk	Yes	197(44.4)	64 (20.5)	261 (34.5)
	No	247(55.6)	248 (79.5)	495 (65.5)
Drunk in the last 12 months	Yes	197(44.4)	64 (20.5)	261(34.5)
	No	247(55.6)	248 (79.5)	495 (65.5)
Current drinkers	Yes	186 (41.9)	62 (19.9)	248(32.8)
	No	258 (58.1)	250 (80.1)	508(67.2)
**Smoking cigarette**				
Ever smoked	yes	61 (13.7)	11 (3.5)	72 (9.5)
	No	383 (86.3)	301 (96.5)	684 (90.5)
Smoked in the last 12months	yes	61 (13.7)	11 (3.5)	72 (9.5)
	No	383(86.3)	301(96.5)	684(90.5)
Current smokers	yes	59(13.3)	11(3.5)	70(9.3)
	No	385(86.7)	301(96.5)	686(90.7)

From a total of 217 khat chewers, 79 (36.4%) chewed occasionally and 38 (17.5%) chewed daily. One hundred seventy three (66.3%) of alcohol drinkers and 24 (33.3%) of cigarette smokers used it occasionally. Similarly, 46 (21.2%) of khat chewers, 41 (15.7%) alcohol drinkers and 6 (8.3%) of cigarette smokers used it once per week. However, 4 (1.6%) of alcohol drinkers and 22 (30.6) of smokers used it on daily bases.

With regard to starting time for psychoactive substances use, 83 (38.2%) of khat chewers and 93 (35.6%) of alcohol drinkers started using while they were preparatory school students. However, 35 (43.2%) of cigarette smokers started it while they were first year university students. Similarly, 16.7% khat chewers, 17.6% alcohol drinkers and 6.2% of cigarette smokers started during high school period. Only very small number of students started khat 3 (1.4%), alcohol 1 (0.4%) and cigarette 1 (1.2%) during third year study period.

The study participants were introduced for khat chewing 176 (81.1%), alcohol drinking 203 (77.8%) and cigarette smoking 60 (83.3%) by peer friends. Similarly, 32 (14.7%) of khat chewers, 42 (16.1%) of alcohol drinkers and 7 (9.7%) of cigarette smokers were introduced by family members.

From 217 study participants who reported ever chewed khat, 80 (40.6%) believed that khat chewing is important to keep alert while reading. Forty-four (20.3%) chewed khat to get relief from stress. On the other hand, 171 (65.5%) of alcohol drinkers used it to relax with friends. Similarly, 27 (10.3%) of the total cigarette smokers used it to get relief from stress.

Five hundred eighty five (77.3%), 608 (80.4%) and 657 (86.9%) of the respondents were aware of problems or complications that could arise from using khat, alcohol and cigarette respectively.

The most commonly mentioned health problems that could arise from khat using were addiction 313 (41.4%) and increase susceptibility to other diseases 199 (26.3%). On the other hand, 397 (52.5%) and 322 (42.6%) of the students knew that lung cancer and addiction are health risks of alcohol drinking and cigarette smoking respectively. Similarly, 197 (26.1%) of the respondents knew that alcohol drinking is a risk factor for liver and heart diseases. Other perceived complications of alcohol drinking mentioned by the respondents include, Gastro intestinal problem 147(19.4%), accidental injury 162 (21.4%) and engaged in unprotected sex 121 (16%).

Table 3 below indicates Socio-demographic and behavioral factors assumed to be associated with khat use among the study participants. Sex, Religion, Ethnicity, original residence, ever drunk alcohol, ever smoked cigarette, family member and peer friends chewed khat and perceived health risk of khat chewing were found to be significantly associated with khat chewing in the last 12 months in the multivariate logistic regression.

**Table 3:** Bivariate and multivariate logistic regression analysis showing Socio-demographic and behavioral correlates of khat chewing within the last 12 months among Axum university students, April 2012

**Variables**	**Chewed**		**COR(95%CI)**	**AOR(95%CI)**
	**Yes**	**No**		
**Sex**				
Male	162	282	2.68(1.89,3.81)	**1.96(1.25, 3.08)**
Female	55	257		
**Religion**				
Orthodox	101	321		
Muslim	76	119	2.03(1.41,2.92)	**3.29(1.92, 5.63)**
Protestant	33	92	1.14(0.72,1.80)	1.39(0.74, 2.65)
Others	7	7	3.18(1.10,9.28)	1.76(0.38, 8.11)
**Ethnicity**				
Tigrie	69	264		
Amhara	80	152	2.01(1.38,2.94)	**2.09(1.26, 3.48)**
Oromo	47	74	2.43(1.55,3.82)	**3.38(1.81, 6.34)**
SNN	21	49	1.64(0.92,2.92)	**2.21(1.02, 4.79)**
**Residence**				
Urban	150	300		
Rural	67	239	0.56(0.40,0.78)	**0.56(0.36, 0.86)**
**Marital status**				**
Single	191	481		
Married	16	42	0.96(0.53,1.75)	
Divorced	5	9	1.40(0.46,4.23)	
Others	5	7	1.80(0.56,5.74)	
**Year of study**				*
1^st^ year	69	209		
2^nd^ year	73	174	1.27(0.86,1.87)	
3^rd^ year	58	139	1.26(0.84,1.90)	
4^th^ year	17	17	3.03(1.47,6.26)	
**Ever drunk alcohol**				
Yes	135	126	5.40(3.84,7.58)	**2.02 (1.20, 3.39)**
No	82	413		
**Ever Smoked cigarette**				
Yes	57	15	12.45(6.86,22.58)	**4.98 (2.47, 10.02)**
No	160	524		
**Family member chewed khat**				
Yes	96	71	5.23(3.63,754)	**1.98(1.24, 3.17)**
No	121	468		
**Peers chewed khat**				
Yes	197	192	17.8(10.88,29.13)	**10.18(5.59, 18.54)**
No	20	347		
**Perceived health risk**				
Yes	154	431	0.61(0.43,0.88	**0.39(0.24, 0.65)**
No	63	108		

** Variables which were not significant in the bivariate analysis.

* Variables which were not significant in the multivariate analysis.

Male students were more likely to chew khat as compared with female students [AOR: 1.96, 95%CI: (1.25, 3.08)]. Compared to Orthodox Christians, being Muslim in religion was strongly associated with khat use [AOR: 3.29, 95%CI: (1.92, 5.63)]. Those students whose friends chew khat were 10.18 times more likely to chew khat as compared to their counterpart [AOR: 10.18, 95%CI: (5.59, 18.54)] (Table 3).

Marital status and perceived health risk of alcohol drinking were not associated with alcohol drinking within the last 12 months in the bivariate logistic regression analysis (P > 0.2).The multivariate logistic regression showed that Sex, religion, ethnicity, ever chewed khat, ever smoked cigarette, family members and peer friends drinks alcohol were found to be significantly associated with alcohol drinking (Table 4).

**Table 4:** Bivariate and multivariate logistic regression showing socio-demographic and behavioral correlates of alcohol drinking within the last 12 months among Axum university students, April 2012

**Variables**	**Drunk alcohol within the last 12 months**		**COR(95%CI)**	**AOR(95%CI)**
	**Yes**	**No**		
**Sex**				
Male	197	247	3.09(2.22,4.31)	**2.12(1.35, 3.32)**
Female	64	248		1
**Religion**				
Orthodox	186	236		1
Muslim	38	157	0.31(0.21,0.46)	**0.14(0.74, 0.25)**
Protestant	30	95	0.40(0.26,0.63)	**0.41(0.21, 0.79)**
Others	7	7	1.27(0.44,3.68)	**0.32(0.03, 1.63)**
**Ethnicity**				
Tigrie	136	197		
Amhara	77	155	0.72(0.51,1.02)	**0.48(0.28, 0.82)**
Oromo	33	88	0.54(0.34,86)	**0.35(0.18, 0.68)**
S/N/N	15	55	0.39(0.21,0.73)	**0.28(0.12, 0.71)**
**Residence**				*
Urban	170	280	1	
Rural	91	215	0.70(0.51,0.95)	
**Marital status**				**
Single	232	440		
Married	20	38	0.99(0.57,1.76)	
Divorced	5	9	1.05(0.35,3.18)	
others	4	8	0.95(0.28,3.18)	
**Year of study**				*
1^st^	74	208		
2^nd^	91	156	1.61(1.11,2.33)	
3^rd^	80	117	1.89(1.28,2.78)	
4^th^	16	18	2.45(1.19,5.06)	
**Ever chewed khat**				
Yes	135	82	5.40(3.84,7.58)	**2.16(1.28, 3.66)**
No	126	413		
**Ever smoked cigarette**				
Yes	64	8	19.78(9.31,42.01)	**8.16(3.35, 19.85)**
No	197	487		
**Family drunk alcohol**				
Yes	111	56	5.80(4.00,8.41)	**2.61(1.56, 4.34)**
No	150	439		
**Peers drunk alcohol**				
Yes	236	153	21.10(13.40,33.23)	**14.00(8.09, 24.24)**
No	25	342		
**Perceived health risk**				**
yes	206	402	0.87(0.60,1.26)	
no	55	93		

** Variables which were not significant in the bivariate analysis.

* Variables which were not significant in the multivariate analysis.

Among the socio demographic and behavioral factors assumed to be associated with cigarette smoking; ethnicity, marital status, year of study and perceived health risk of cigarette smoking were not significantly associated with cigarette smoking within the last 12 months in the bivariate logistic regression. On the other hand, the multivariate logistic regression showed that sex, religion, ever chewed khat, ever drunk alcohol, and peer friends smoked cigarette were found to be significantly associated with cigarette smoking within the last 12 months (Table 5).

**Table 5:** Bivariate and multivariate logistic regression, showing Socio-demographic and behavioral correlates of cigarette smoking within the last 12 months among Axum university students, April 2012

**Variables**	**cigarette use within the last 12 months**		**COR(95%CI)**	**AOR(95%CI)**
	**Yes**	**No**		
**Sex**				
Male	61	383	4.36(2.25,8.43)	**2.60(1.17,5.76)**
Female	11	301		
**Religion**				
Orthodox	45	377		
Muslim	12	183	0.55(0.28,1.06)	0.76(0.34, 1.72)
Protestant	9	116	0.65(0.31,1.37)	0.80(0.32, 1.99)
Others	6	8	6.28(2.09,18.93)	**7.93(1.67, 37.62)**
**Ethnicity**				*
Tigrie	23	310		
Amhara	30	202	2.00(1.13,3.54)	
Oromo	13	108	1.62(0.79,3.32)	
S/N/N	6	64	1.26(0.49,3.23)	
**Residence**				*
Urban	51	399		
Rural	21	285	0.58(0.34,0.98)	
**Marital status**				**
Single	65	607		
Married	3	55	0.51(0.16,1.67)	
Divorced	2	12	1.56(0.34,7.11)	
others	2	10	1.87(0.40,8.71)	
**Year of study**				**
1^st^	22	256		
2^nd^	23	224	1.20(0.65,2.20)	
3^rd^	23	174	1.54(0.83,2.85)	
4^th^	4	30	1.55(0.50,4.81)	
**Ever chewed khat**				
Yes	57	160	12.45(6.86,22.58)	**4.90(2.36,9.73)**
No	15	524		
**Ever drunk alcohol**				
Yes	64	197	19.79(9.31,42.01)	**6.54(2.66,16.05)**
No	8	487		
**Family smoke cigarette**				*
Yes	38	142	2.03(1.21,3.41)	
No	34	542		
**Peers smoke cigarette**				
Yes	69	320	26.16(8.16,83.93)	**4.61(1.26,16.78)**
No	3	364		
**Perceived health risk**				**
yes	66	6	1.73(0.73,4.11)	
no	591	93		

** Variables which were not significant in the bivariate analysis.

* Variables which were not significant in the multivariate analysis.

## Discussion

This study showed that there is substantial burden of psychoactive substances use among undergraduate students in Axum University. The overall life time and current prevalence of psychoactive substances use among Axum University students were 45.9% and 44.8% respectively. These findings were significantly lower than a similar study done among high school students in Nigeria, in which the life time and current prevalence were 87.3% and 69.2% respectively. These findings were also lower than a similar study done among college students in Eldoret, Western Kenya where the lifetime prevalence rate of any substances use was 69.8% [10].

The possible explanation for the observed differences in substances use could be due to the differences in knowledge on the health risks of substances use. For instance, the study among high school students in Nigeria showed that 73% of the study subjects were not aware of the health risks associated with substances use, whereas more than 70% of our study subjects were aware of the health risks of substances use.

Our findings indicated that life time and current khat use was 28.7% and 27.9% respectively. These findings were consistent with a study done among college students in North West Ethiopia, in which the life time prevalence of khat chewing was 26.7%. Similarly, these findings were in line with studies done among college and high school students of Jazan region, Saudi Arabia, among staffs of Jimma University and study done in Jimma town with prevalence rate of 21%, 30.8% and 30.6% respectively [12,14,15]. However, our findings were higher than study done among undergraduate medical students in Addis Ababa University with the past year and current prevalence of 7% and 4% respectively [4]. A similar study in Somalia revealed that the life time prevalence of khat chewing was 81.6% among men and 43.3% among women, which is higher than the findings of this study where only 36.5% males and 17.6% females chewed khat. This difference could be due to the study in Somalia were composed of individuals reportedly under severe stress and in a context of social disruption which may potentially increases substances use [16].

Our findings indicated that life time and current alcohol use was 34.5% and 32.8% respectively. These results were lower than a studies done at Federal University of Alagoas, Brazil and among college students in Eldoret, Western Kenya with alcohol consumption rate of 87.6% and 51.9% respectively [10,17].

Similarly, our reported rate of alcohol consumption was lower than a study done among adolescents in rapidly developing countries with prevalence of 49% and 48% for male and female respectively [18]. However, our reported life-time alcohol consumption was higher than a study done among medical students in Addis Ababa University 22% [4].

Our findings indicated that life time and current cigarette smoking was 9.5% and 9.3% respectively. The reported rate of cigarette smoking in this study was almost consistent with study conducted among college students in North West Ethiopia where the life time and current prevalence were 13.1% and 8.1% respectively [12]. Our findings were also in agreement with community based study conducted in Butajira town, among adolescents in Ethiopia and among undergraduate medical students of Addis Ababa University in Ethiopia [4], [19], [20]. This finding was also consistent with a study conducted among undergraduate students in University of IIorion, Nigeria in which the current prevalence of smoking was 5.7% [21]. However, the results of this study were lower than a studies conducted among adolescents in rural Zambia, among High school students in Harare, Zimbabwe and among college students in Eldoret, Western Kenya with prevalence of cigarette smoking 27%, 28.8% and 42.8% respectively [10,22-24].

The mean age at which the respondents started khat chewing, alcohol drinking and cigarette smoking was 20.1 years ± 2.75 sd, 19.5 years ± 2.2 sd and 20.5 years ± 2.2 sd respectively, This finding was slightly higher than a study done among college students in North west Ethiopia in which the mean age for starting khat chewing and cigarette smoking was 17.3 and 18.2 years, respectively [12]. The reported mean age for khat chewing was also significantly higher than study done in Eastern Ethiopia in which the mean age was 15.1 ± 2.33 years. This could be explained by the fact that the cultivation and consumption of khat is practiced widely in the Eastern Ethiopia and it is more a part of the culture than that of our study.

Our reported starting time for psychoactive substances use was almost consistent with study conducted in North West Ethiopia, where majority of the lifetime smokers 31.7 % and of the lifetime khat chewers 45.6 % started smoking and chewing while they were senior secondary school students followed by 1^st^ year college [12]. This indicates late high school and freshman university students are at higher risk for experimentation of psychoactive substances use.

A large proportion of the study participants were introduced for khat chewing 81.1%, alcohol drinking 77.8% and cigarette smoking 83.3% by their peer friends. This finding was supported by a study conducted among college students in Eldoret, Western Kenya where about 75.1% of the students were introduced by their peer friends [10].

Being male, Muslim in religion, Oromo Ethnic group and from urban back ground was significantly and positively associated with khat chewing within the last 12 months. This might be due to the fact that khat growing and the practice of chewing have traditionally been confined to some places where Muslim populations were found. Similarly, khat cultivation could be high in some areas of Oromia region and those students from urban origin might have greater access to khat than students from rural origin. This study is supported by a study conducted among high school students of Jazan region [14].

Ever alcohol use, cigarette use, family member chew khat, peer friends chew khat were strongly and positively associated with khat chewing within the last 12 months. These findings were consistent with study done among staff of Jimma University which showed that being male, Muslim and habit of alcohol and cigarette intake had significant association with khat chewing [15]. Our reported findings were also supported by similar study done among students in Jimma University and Eastern Ethiopia where majority of khat users were male, Muslim and Oromo in ethnicity [13,15].

Being male, belonging to Orthodox Christianity, Tigre in ethnicity, ever chewed khat, ever smoked cigarette, family member and peer friends alcohol use were independent predictors of alcohol drinking among the study subjects.

These findings were in line with similar study done among medical students in Addis Ababa University and in rapidly developing countries [4,18].

With regard to cigarette smoking, there was a statistically significant difference in cigarette smoking between males and females, with males having a higher rate than females [AOR: 2.597, 95%CI: (1.17, 5.76)]. This finding was consistent with what has been found in other studies [4,10,12,24]. Alcohol and khat use was strongly associated with cigarette smoking within the last 12 months. Similarly, having smoker friends has been strongly associated with cigarette smoking. The findings of this study were consistent with similar studies done in Zimbabwe, Zambia and Nigeria where smoking has been associated with gender, having friends that smoke and alcohol use [21,23,24].

This study had some limitations; first, the study used a descriptive cross-sectional design that cannot establish trends and causality between substances use and potential risk factors. Second, the data was collected based on self-report of the students and may be subjected to recall bias and under-reporting of substances use due to social desirability bias. Third, findings from this study may not be generalized to the whole young people, because the study involved only university students.

## Conclusion

This study revealed that psychoactive substances use became an urgent problem among undergraduate university students, with the most commonly used substances being alcohol, khat and cigarette respectively. Most of the students started substances use during their preparatory school and first year university study period and the most commonly mentioned reason for khat, alcohol and cigarette use among university students were to keep alert while reading, for relaxation with friends and to get relief from stress respectively. More than three-quarter of the respondents were aware of the complications that could arise from their substances use, though the prevalence is still high. Peer friends and family member substances use were identified to be the most significant factors for psychoactive substances use by the students.

### Recommendation

The University need to prepare students involved open forums and conferences to create understanding on the ill effects of psychoactive substances use and to bring behavioral change in collaboration with other organizations. The ministry of Education need to focus and Integrate education about substances use into the curricula of primary and secondary schools. Peer educators need to be established and strengthened in all universities, high schools and preparatory schools; because involvement of peers and role models would have a high probability of success by providing education about substances and its effects in a friendly manner. Universities need to teach and monitor their students with special focus on fresh man students and Universities substances control efforts need to target on the environment where substances are found, parents and students.

## Competing interests

The authors declare that they have no competing interests.

## Authors’ contributions

MG conceived the original idea, involved in proposal writing, designed the study and participated in all implementation stages of the project. MG also analyzed the data and finalized the write up of the manuscript. AF was responsible for critically revising the proposal and the manuscript, and participated in its design and interpretation. TF was responsible for data collection, initial analysis and drafting of manuscript. All authors reviewed and approved the final manuscript.

## Pre-publication history

The pre-publication history for this paper can be accessed here:

http://www.biomedcentral.com/1471-2458/13/693/prepub

## References

[B1] Odejide A Status of drug use/abuse in Africa Int J Mental Health 2006 4 87 102

[B2] WHO Global status report on alcohol and health 2011 XI 5

[B3] WHO Report On THE Global Tobacco Epidemic 2011 7 74

[B4] Deressa W Azazh A Substances use and its predictors among undergraduate medical students of Addis Ababa University in Ethiopia BMC Public Health 2011 11 660 10.1186/1471-2458-11-660 21859483 PMC3170623

[B5] Oshodi O Aina O Onajole A Substances use among secondary school students in an urban setting in Nigeria: prevalence and associated factors African J Psychiatry 2010 13 32 57 10.4314/ajpsy.v13i1.53430 20428599

[B6] Grossbard JR Mastroleo NR Kilmer JR Lee CM Turrisi R Larimer ME Substances Use Patterns Among First-Year College Students:Secondary Effects of a Combined Alcohol Intervention J Subst Abuse Treat 2010 39 4 384 390 10.1016/j.jsat.2010.07.001 20817383 PMC2967640

[B7] Kebede Y Abula T Ayele B Feleke A Degu G Kifle A Substances Abuse For the Ethiopian Health Center Team Ethiop Public Health Train Initiative 2005 81

[B8] Joseph A Depression, Substances Abuse and College Student Engagement: A Review of the Literature 2003

[B9] EPHA Emerging Public Health Problem in Ethiopia, Annual conference of The Ethiopian public Health association 2006 Addis Ababa: EPHA

[B10] Atwoli L Mungla PA Ndung’u MN Kinoti KC Ogot EM Prevalence of substances use among college students in Eldoret, western Kenya BMC Psychiatry 2011 11 34 10.1186/1471-244X-11-34 21356035 PMC3053226

[B11] Fekadu A Alem A Hanlon C Alcohol and Drug Abuse in Ethiopia African J Drug Alcohol Studies 2007 6 1 40 47

[B12] Kebede Y Cigarette smoking and Khat chewing among college students in North West Ethiopia Ethiop J Health Dev 2002 16 1 9 17

[B13] Reda AA Moges A Biadgilign S Wondmagegn BY Prevalence and Determinants of Khat (Catha edulis) Chewing among High School Students in Eastern Ethiopia: A Cross-Sectional Study PLoS One 2012 7 3 2 4 10.1371/journal.pone.0033946 PMC3316517 22479484

[B14] Ageely HM Prevalence of Khat chewing in college and secondary (high) school students of Jazan region Saudi Arabia Harm Reduction J 2009 6 11 10.1186/1477-7517-6-11 PMC2707373 19545389

[B15] Gelaw Y Haile-Amlak A Khat chewing and its socio-demographic correlates among the staff of Jimma University EthiopJHealth Devt 2004 18 3 6 15

[B16] Odenwald M Khat use as a risk factor for psychotic disorders in Somalia Bio Med Central Med 2005 3 1 5 9 10.1186/1741-7015-3-5 PMC554104 15707502

[B17] Silva Y Brandão T The prevalence of alcohol consumption among the students newly enrolled at a public university J Pharm Bioallied Sciences 2011 3 3 345 349 10.4103/0975-7406.84434 PMC3178940 21966154

[B18] Faeh D Viswanathan B Chiolero A Warren W Bovet P Clustering of smoking, alcohol drinking and cannabis use in adolescents in a rapidly developing country BMC Public Health 2006 6 169 10.1186/1471-2458-6-169 16803621 PMC1564395

[B19] Schoenmaker N Hermanides J Davey G Prevalence and predictors of smoking in Butajira town Ethiop EthiopJHealth Dev 2005 19 3 182 187

[B20] Rudatsikira E Abdo A Muula AS Prevalence and determinants of adolescent tobacco smoking in Addis Ababa Ethiop BMC Public Health 2007 7 176 1 6 10.1186/1471-2458-7-176 PMC1940247 17651482

[B21] Fawibe A Shittu A Prevalence and characteristics of cigarette smokers among undergraduates of the University of Ilorin Nigeria Niger J Clin Pract 2011 14 2 201 205 10.4103/1119-3077.84016 21860140

[B22] Rapeah M Munirah Y Latifah O Faizah K Norsimah S Maryana M Factors influencing smoking behaviors among male adolescents in Kuantan district Annual Dent Univ Malaya 2009 15 2 77 81

[B23] Siziya S Rudatsikira E Muula A Ntata P Predictors of cigarette smoking among adolescents in rural Zambia Rural Remote Health 2007 7 728 17900223

[B24] Bandason T Rusakaniko S Prevalence and associated factors of smoking among secondary school students in Harare Zimbabwe Tob Induced Dis 2010 8 12 10.1186/1617-9625-8-12 PMC2984581 20979604

